# Capillary-force-induced collapse lithography for controlled plasmonic nanogap structures

**DOI:** 10.1038/s41378-020-0177-8

**Published:** 2020-09-21

**Authors:** Inki Kim, Jungho Mun, Wooseup Hwang, Younghwan Yang, Junsuk Rho

**Affiliations:** 1 Department of Mechanical Engineering, Pohang University of Science and Technology (POSTECH), Pohang, 37673 Republic of Korea; 2 Department of Chemical Engineering, Pohang University of Science and Technology (POSTECH), Pohang, 37673 Republic of Korea; 3 Department of Chemistry, Pohang University of Science and Technology (POSTECH), Pohang, 37673 Republic of Korea; 4 National Institute of Nanomaterials and Technology (NINT), Pohang, 37673 Republic of Korea

**Keywords:** Nanophotonics and plasmonics, Micro-optics, Structural properties

## Abstract

The capillary force effect is one of the most important fabrication parameters that must be considered at the micro/nanoscale because it is strong enough to deform micro/nanostructures. However, the deformation of micro/nanostructures due to such capillary forces (e.g., stiction and collapse) has been regarded as an undesirable and uncontrollable obstacle to be avoided during fabrication. Here, we present a capillary-force-induced collapse lithography (CCL) technique, which exploits the capillary force to precisely control the collapse of micro/nanostructures. CCL uses electron-beam lithography, so nanopillars with various shapes can be fabricated by precisely controlling the capillary-force-dominant cohesion process and the nanopillar-geometry-dominant collapse process by adjusting the fabrication parameters such as the development time, electron dose, and shape of the nanopillars. CCL aims to achieve sub-10-nm plasmonic nanogap structures that promote extremely strong focusing of light. CCL is a simple and straightforward method to realize such nanogap structures that are needed for further research such as on plasmonic nanosensors.

## Microlithography: Pioneering lithography technique for fabricating plasmonic nano-gap structures

An innovative technique—capillary-force-induced collapse lithography (CCL)—has been developed to easily fabricate plasmonic nano-gap structures, which with a nano-gap of <10 nm are able to compress light and dramatically increase the intensity of electric fields. Deformation of nanostructures due to capillary forces (e.g., collapse) has been a considerable obstacle in fabricating plasmonic nano-gap structures. However, a team headed by Junsuk Rho at Pohang University of Science and Technology (POSTECH), Republic of Korea developed CCL, which exploits capillary forces to precisely control the collapse of the nanostructures. CCL allows the easy fabrication of sub-10-nm plasmonic nano-gap structures, which are difficult to achieve with conventional lithography. The authors believe that their simple, straightforward CCL technique will pave the way toward nano-manufacturing technology that enables the effective production of sub-10-nm plasmonic nano-gap structures on demand.

## Introduction

The capillary phenomenon occurs when a fluid flows through a narrow space and is often observed in nature^1–5^. At the micro/nanoscale, this force can easily deform micro/nanostructures^6–9^, making it one of the most important process conditions during micro/nanoscale fabrication. However, the deformation of micro/nanostructures due to capillary forces, such as stiction and collapse, has not been controllable and has therefore been undesirable. To overcome these shortcomings, many researchers have suggested ways to use capillary forces to control the collapse or self-assembly of nanostructures^10–15^. One example is a capillary-force-induced nanocohesion method to make hierarchical nanostructures at the 10-nm scale^12^. The collapse of nanopillars has been reported^13^, and recent research has developed sub-10-nm nanogap structures by exploiting the collapse of such nanopillars^14,15^.

Sub-10-nm nanogap structures can be used to compress light into deep subwavelength volumes and, as a result, drastically increase the intensity of the electric fields in the nanogap region. Initially, realization of such sub-10-nm nanogap structures in a top-down fabrication manner required the use of tedious and expensive nanofabrication processes such as atomic layer deposition^16–18^, focused ion beam milling^19–21^ (FIB), and electron beam lithography^22–24^ (EBL). Recently, however, unconventional nanogap structure fabrication methods based on collapse have been reported. For instance, collapsible nanopillar structures have been obtained using nanoimprints, where two adjacent nanopillars collapse toward each other and form a controlled nanogap^14,15^. Given the advantages of the collapse approach, such as cost effectiveness and high reproducibility, these straightforward collapse approaches can be used as an alternative to conventional beam-based lithography techniques. Particularly, the collapse approach has a higher yield (or reproducibility) compared with the EBL and FIB processes, as they require highly specific fabrication conditions such as the substrate type, dose, pattern density, and proximity effect. Until now, the shape of the nanogap structures that can be realized by collapse-based lithography has been limited compared with the EBL and FIB methods.

Here, we propose a capillary-force-induced collapse lithography (CCL) technique that can precisely control the collapse of structures similar to how dominos fall and can be used to realize sub-10-nm plasmonic nanogap structures. We exploit the cohesion process between nanopillars and the collapse effect of each column to finely control the collapse of structures with sizes from <50 nm to 1 µm. This technique uses EBL, so a sub-10-nm bowtie- or flower-shaped gap structure can be realized. CCL can be an alternative way to create an ordered single-nanogap or multiple-nanogap structure with desired optical properties. In this paper, we introduce the experimental procedure for realizing sub-10-nm plasmonic nanogap structures and conduct full-wave electromagnetic simulations to demonstrate the confined electric field in the nanogap region. Furthermore, surface-enhanced Raman scattering (SERS) measurements are performed on the bowtie-shaped nanogap platform to prove the large field enhancement in the sub-10-nm gap region.

## Results and discussion

### Working mechanisms of CCL

**Fig. 1:**
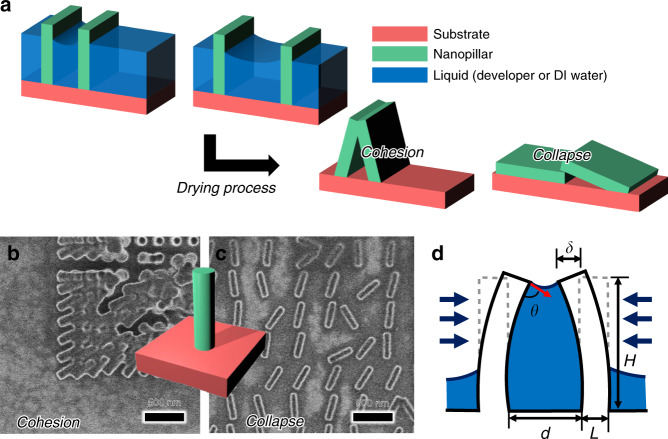
Capillary-force-induced collapse lithography (CCL) mechanism. **a** Basic idea of CCL, cohesion and collapse. **b**, **c** Scanning electron microscope (SEM) images of fallen nanopillars 300 nm in height and 45 nm in diameter. **d** Schematic diagram of a nanopillar for sway model analysis

CCL combines the concepts of cohesion and collapse to realize precise patterning (Fig. 1a). Cohesion occurs when the nanostructures are arranged densely and the capillary force is strong enough to deform them, i.e., the cohesion process is driven by the capillary force. Due to the high capillary force of the fluid interposed between adjacent nanostructures, a mechanical deformation, and corresponding aggregation occur. Collapse occurs even if the nanostructures are not closely arranged. The capillary force is weaker than that necessary for cohesion but is influenced more by the geometrical properties of the nanopillars than cohesion is, so the collapse process is driven by the geometric properties. For the collapse effect, since the forces acting on the structure depend on the direction of the fluid spread or the direction of drying in the development process, the shape of each structure is the dominant factor determining the collapse direction. If the nanopillars are closely arranged, then the cohesion phenomenon causes structures to collapse in the form of self-assembly. If the pillars are not arranged closely, then the collapse effect causes random falling of nanostructures (Fig. 1b, c).

Here, we use the beam sway model to describe the nanopillar collapse mechanism, where the resist is considered an elastic body and the pillar structure is assumed to be a beam supported at one end^25,26^ (Fig. 1d). The cohesive pressure (or capillary pressure) *P* acting on the nanopillar can be expressed as

1 $$P = 2\sigma \cos \theta /d$$

where *σ* is the surface tension of the liquid, *θ* is the contact angle between the liquid and solid structure, and *d* is the distance between two nanopillars. The deflection of a nanopillar *δ*, cross-sectional second moment *I* (for a square pillar) and force per unit length *w* are expressed as

2 $$\delta = wH^4/8EI$$

3 $$I = DL^3/12$$

4 $$w = PD$$

where *H* is the pattern height, *L* is the pattern width, *D* is the pattern depth, *w* is the force per unit length, and *E* is the Young’s modulus. The cohesive force per unit length *P*_1_ is expressed as

5 $$P_1 = 2\sigma D\cos \theta /(d - 2\delta ) + 8\sigma D\sin \theta \cdot \delta /3H(d - 2\delta )$$

where *P*_1_ acts on the resist pattern when *θ* = 90°. The restoring force per unit length *F*_1_ can be expressed as

6 $$F_1 = (2DL^3/3H^4)E\delta$$

When *P*_1_ is greater than *F*_1_, the resist pattern collapses. Therefore, to effectively collapse the nanostructures, the restoring force should be sufficiently small. This can be achieved by increasing the aspect ratio and decreasing the Young’s modulus of the structures. Particularly, the critical Young’s modulus where fracture occurs can be expressed as

7 $$E = 24\sigma H^4/L^3d^2$$

The CCL technique uses cohesion and collapse effects simultaneously for two reasons. First, the structure consists of very densely arrayed nanostructures, so arbitrary control of the shape of the nanopillars is a difficult task, making it almost necessary to fabricate cylindrical nanopillars. Therefore, to realize nanopillars with various shapes, the collapse effect should be enhanced, while the spatial density of the structure should be decreased. Second, when the nanopillars are not cylindrical, the contact area between the substrate and the column may be somewhat increased; in this case, even if the shape of the nanopillars is suitable for producing a collapse effect (e.g., an asymmetric geometry), the restoring force is so large that cohesion is needed to help collapse the structure.

### Fabrication variables in CCL

**Fig. 2:**
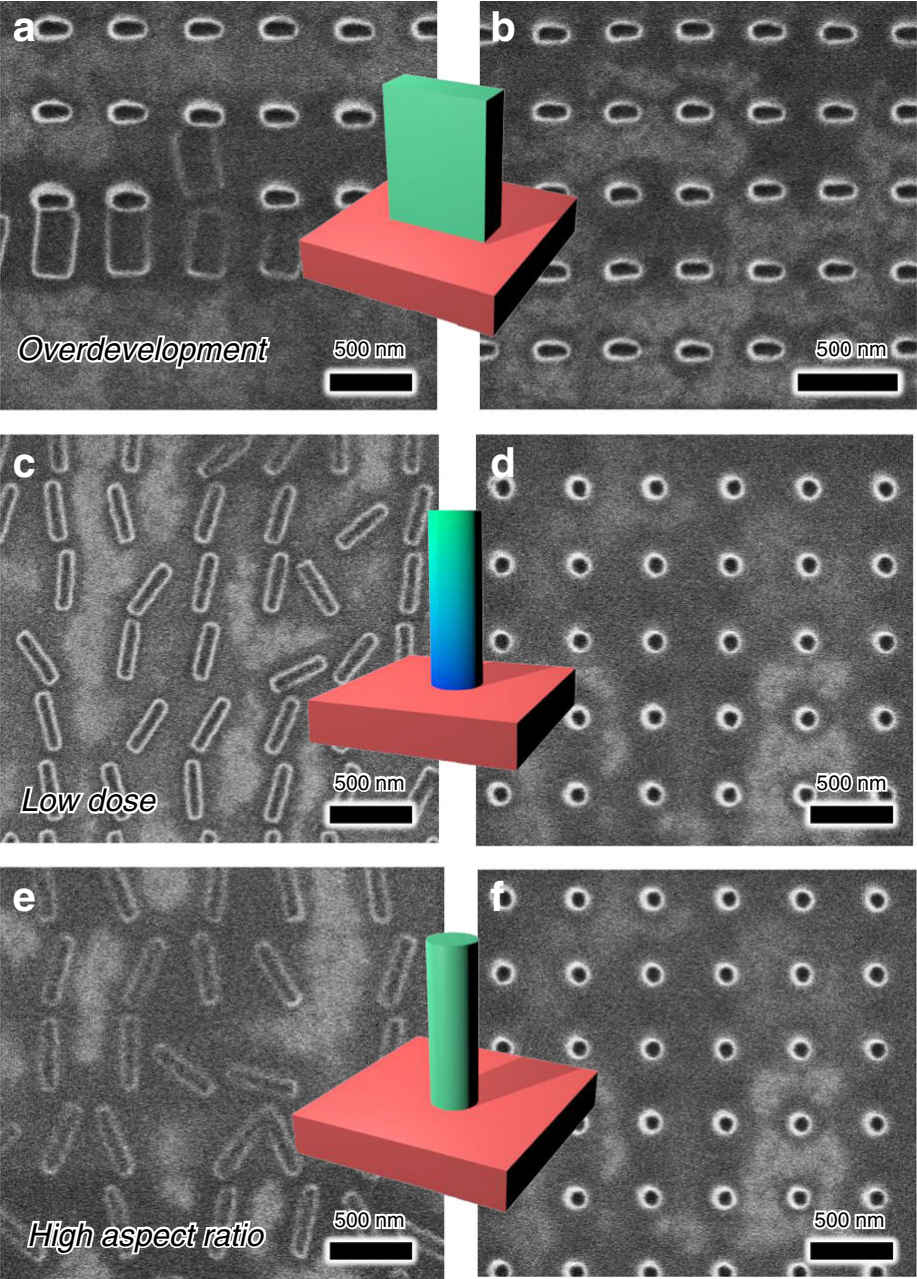
Various fabrication variables in CCL. **a**, **b** SEM images of 70 nm × 210 nm rectangular nanopillars subject to different development times: **a** 100 s development; **b** 80 s development. **c**, **d** SEM images of cylindrical nanopillars with a 70-nm diameter fabricated using different electron doses: **c** 320 µC/cm^2^; **d** 800 µC/cm^2^. The gradient-colored column shows that the bottom part of the cylinder is less likely to cross-link. **e**, **f** SEM images of cylindrical nanopillars with different aspect ratios *A*: **e***A* = 6.7 (diameter = 45 nm); **f***A* = 4 (diameter = 70 nm)

In this section, we introduce the effects of various process variables of the EBL to control the nanopillar collapse. The development time *t*_D_, electron beam dose (energy) *D*, and structure characteristics of the column are particularly important variables (Fig. 2). Particularly, in terms of the restoring force for the collapse effect, the development and dose conditions are related to the Young’s modulus of the nanostructures and to the nanostructure geometry in terms of the aspect ratio. The basic EBL process is as follows. To define the nanopillar structure, we use a 300 nm layer of novolac-based resist (AR-N 7520.11) as a negative-tone electron-beam (e-beam) resist. The AR-N resist is coated on a silicon substrate, and the assembly is subjected to an electron beam with an energy of ~240–960 µC/cm^2^. Then, we use an AR-300 47 developer and rinsing with deionized water to develop the exposed area.

To quantify the effect of *t*_D_, we made rectangular pillars with 70 nm vertical and 210 nm horizontal widths by setting *D* = 320 µC/cm^2^. One sample was developed for 80 s, and the other was developed for 100 s. The sample treated with *t*_D_ = 100 s collapsed easily. Of course, *t*_D_ and the other development conditions used in this experiment may differ slightly depending on the shape of the column to be fabricated, but a structure that has been overdeveloped generally collapses easily (Fig. 2a, b).

Then, we investigated how *D* affects the collapse. To produce a cylinder with a diameter of 70 nm, one sample was exposed to *D* = 320 µC/cm^2^ and another to *D* = 800 µC/cm^2^, and then, both samples were developed for 80 s. The first sample collapsed easily, and the direction of collapse was random. The electron beam-irradiated portion of the negative resist forms bonds or cross-links, and the bottom part of the cylinder is less likely to cross-link when it is exposed to a weak dose than when it is exposed to a strong dose. As a result, the elastic modulus of the nanopillars differs depending on the dose, and the Young’s modulus is generally lower when the dose is weaker^27^. In turn, the restoring force is lower, and collapse occurs more easily after a weak dose compared with a strong dose (Fig. 2c, d).

The aspect ratio *A* and symmetry of the nanopillar should also be considered. First, to determine how the aspect ratio affects the collapse of the nanopillars, cylinders were fabricated with *A* = 6.7 (diameter = 45 nm) or *A* = 4 (diameter = 70 nm). To prevent collapse due to the development time and dose conditions, we set *t*_D_ = 80 s and *D* = 800 µC/cm^2^. Pillars with *A* = 4 did not collapse, and pillars with *A* = 6.7 did (Fig. 2e, f).

**Fig. 3:**
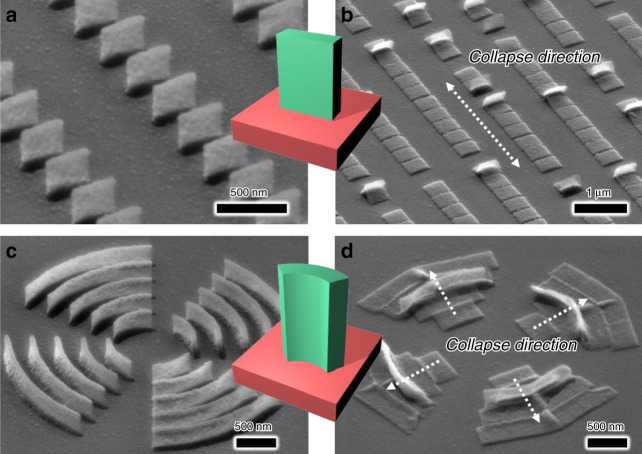
Collapse effect under an asymmetric geometry. **a** SEM images of asymmetric rectangle-shaped nanopillars before collapse and **b** after collapse. **c** SEM images of asymmetric curved nanopillars before collapse and **d** after collapse. With the help of nanocohesion between adjacent nanopillars and asymmetric shapes, the collapse direction can be precisely controlled

Asymmetric shapes can be used to precisely control the direction of collapse, whereas *t*_D_, *D*, and *A* can be used to effectively cause the collapse of nanostructures. One way to maximize the asymmetry is to create a long rectangular column lying on one side. We fabricated 50 nm × 400 nm rectangular pillars. Due to the asymmetry of the rectangles, the pillars collapsed in a direction perpendicular to the longer edge (Fig. 3a). However, they could fall in one of two directions (Fig. 3b), so an additional asymmetric element was needed to make the structure collapse in only one direction. Therefore, we fabricated curved fin-shaped nanopillars (Fig. 3c) with sizes from 500 nm to 2 µm or more, which collapsed in the direction of curvature (Fig. 3d).

The fabrication conditions (*t*_D_, *D*, and geometric characteristics) introduced in this section can be varied depending on the shape that the user wants to realize. These fabrication conditions must be considered for any form of nanopillar collapse. Furthermore, accurate calculation of mechanical properties, such as the capillary force and restoring force, can enable the nanopillar shape and collapse dynamics to be realized in a variety of designs and directions.

### Sub-10-nm plasmonic nanogap structures fabricated by CCL

In this section, we introduce a CCL method to fabricate sub-10-nm plasmonic nanogap structures. In a sub-10-nm gap region, light is strongly focused, and various physical phenomena, such as surface-enhanced Raman scattering^28^, nonlinear optics^29^, light generation^30^, and quantum plasmonics^31^, can be observed. Recently, several studies have realized nanogap structures using a collapse effect, but the shapes of these structures (and corresponding optical properties) are limited by the shape of the pillars. However, CCL can manipulate the optical characteristics of focused light by diversifying the shapes of nanopillars.

**Fig. 4:**
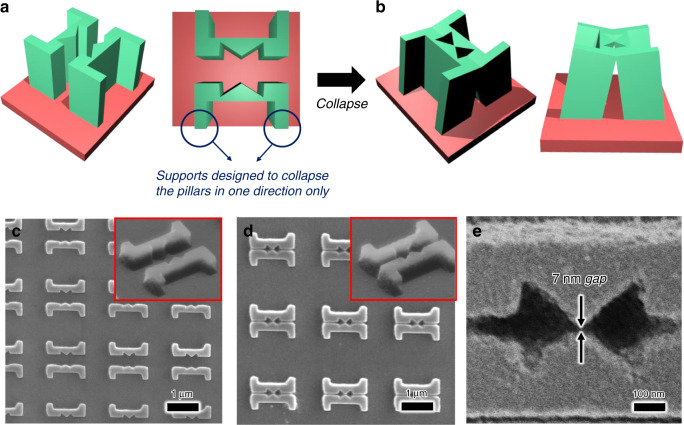
Bowtie nanogap structures fabricated by CCL. **a**, **b** Nanopillar design and collapse mechanism to achieve bowtie nanogap structures. **c** SEM image of unfallen nanopillars. **d** SEM image of fallen nanopillars. **e** Sub-10-nm gap structure with a bowtie shape

In this study, a bowtie-type nanogap was fabricated to realize a single-nanogap structure. The bowtie structure can efficiently focus light in the center of two opposing triangular-shaped openings. The resonance wavelength of the focused light can be tuned by adjusting the angles or sizes of the two triangles. The pillars that face each other were carved into half-bowtie shapes so that when the nanopillars collapse, they form a bowtie. Behind the columns, we also built a support structure that would collapse so that the columns facing each other would collapse effectively (Fig. 4a, b). The important consideration here is that the restoring force of the nanopillar is large because the size of each column is >1 µm. Because of this restoring force, collapse requires a large capillary force acting between the two pillars. For this purpose, the distance between the two pillars was set to 100 nm. If the distance is >100 nm, then the columns do not fall (Fig. 4c, d). By adjusting these conditions to collapse the nanopillars, a bowtie-type nanostructure with a sub-10-nm gap was formed. To induce a plasmonic effect, the photoresist structure was collapsed and then coated with a 10-nm-thick gold thin film (Fig. 4e).

**Fig. 5:**
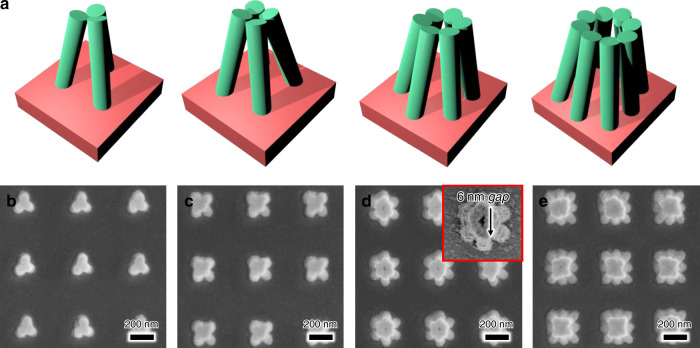
Flower-shaped nanogap structures fabricated by CCL. **a** Schematic of flower-shaped nanogap structures. The structures are designed to form *N* nanogaps when the *N* columns fall over each other. SEM images of flower structures with **b** three, **c** four, **d** six, and **e** eight nanogaps. The nanogap size is ~6 nm (inset of **d**)

To form multiple nanogaps, we devised nanopillar arrays that form a flower shape when they collapse. The nanopillar arrays were designed to form *N* nanogaps when the *N* columns fall over each other. A nanogap is formed at the contact area where the pillars contact each other, and a nanogap is realized between the petals. We observed nanostructures formed by the collapse of three, four, six or eight nanopillars with a diameter of 45 nm. The nanogaps formed between the petals were <10 nm, and light could be strongly focused at each point (Fig. 5).

**Fig. 6:**
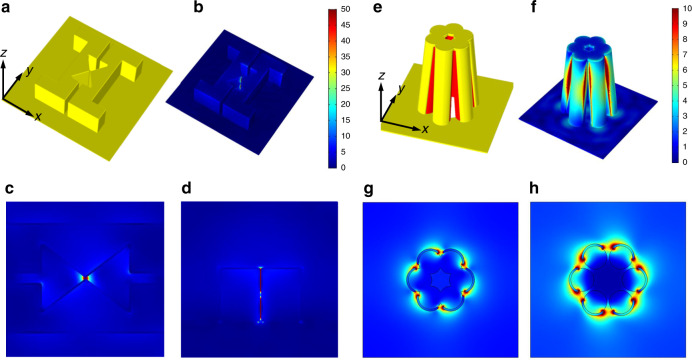
3D full-wave electromagnetic simulation. An unpolarized light source incident on the structure in the −*z* direction was used, and the electric field distribution at 900 nm was calculated. The refractive index of the nanopillars is 1.6, and Palik’s experimental value was used for the gold thin film^32^. **a** 3D schematic of the bowtie-shaped structure. The yellow color represents the covering gold thin layer. **b** 3D electric field distribution of the bowtie-shaped structure. **c***xy* plane cross-section view at *z* = 225 nm. **d***xz* plane cross-section view at the center. **e** 3D schematic of the flower-shaped structure (the red-colored part represents photoresist and the yellow-colored part represents the covering gold thin layer). **f** 3D electric field distribution of the flower-shaped structure. **g***xy* plane cross-section view at *z* = 275 nm (top of the pillar). **h***xy* plane cross-section view at *z* = 140 nm (middle of the pillar)

A full-wave electromagnetic simulation based on the finite element method (FEM) was performed to verify the tightly and strongly focused electric field around the sub-10-nm nanogap structures (Fig. 6). The simulations were carried out using the commercial FEM solver COMSOL Multiphysics, and the structures were modeled as close as possible to the scanning electron microscope (SEM) images to obtain accurate results. We simulated bowtie-shaped nanopillars producing a single nanogap and flower-shaped nanopillars producing multiple nanogaps. Each column was covered with a 10-nm-thick gold film, and the refractive index of the photoresist was fixed to 1.6, as indicated in the manufacturer’s product manual (Allresist). First, the bowtie-shaped nanopillars forming a single nanogap structure can strongly squeeze the electric field in the central part where the triangles meet each other (Fig. 6a–d). In addition, as shown in Fig. 6d, the *xz* plane cross-section view at the center shows that the electric field is strongly focused to the bottom of the column. Similarly, in the flower-shaped nanostructure that forms multiple nanogaps, the electric field is strongly focused in the nanogaps between the columns (Fig. 6e–h). Because the resonance wavelength and spectrum of the light focused at each nanogap depends on the geometry of the structure, they can be tailored to the user’s purpose.

**Fig. 7:**
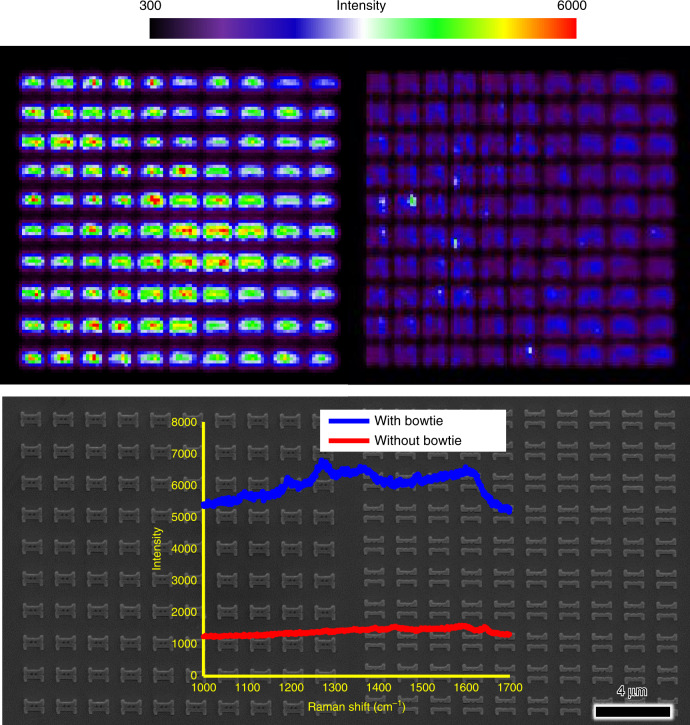
2D maps of the SERS intensity of the 1365 cm^−1^ band of R6G. The mapping pixel size is 200 nm × 200 nm. Lower part: SEM image for the corresponding area where SERS experiments were conducted, and SERS spectra from rhodamine 6G on the bowtie structures

Along with the full-wave electromagnetic simulation, we conducted a SERS measurement to show the large field enhancement and confinement in the sub-10-nm bowtie structures. The probe molecule rhodamine 6G (R6G) was diluted in 10^−8^ M water, and the solution was dropped and dried on the sample. The bowtie structure with a sub-10-nm gap showed clear SERS signals, while the noncollapsed nanostructures showed low SERS signals because of hotspot absence (Fig. 7). Therefore, the sub-10-nm nanogap structures are expected to be applicable to ultrahigh sensitivity nanosensors.

### Conclusion

We have demonstrated a pioneering capillary-force-induced collapse lithography (CCL) technique. CCL exploits a collapse phenomenon to easily fabricate sub-10-nm plasmonic nanogap structures, which are difficult to be realized by conventional lithography. In this study, fabrication parameters that affect the cohesion and collapse of nanopillars were closely investigated, and a method of effective collapse of nanopillars was introduced. Furthermore, CCL was used to generate nanogap structures shaped like bowties or flowers by using various nanopillar designs. The strongly focused light at the sub-10-nm gap area, which was confirmed by full-wave electromagnetic simulations and SERS measurements, could be utilized in extreme applications such as single-molecule SERS platforms, optical tweezers and single-photon sources. The CCL technique will pave the way toward nanomanufacturing technology that enables the effective production of sub-10-nm plasmonic nanogap structures on demand.

## Materials and methods

### Device fabrication

The device fabrication began on a 500-µm-thick silicon substrate. The negative photoresist (Allresist, AR-N 7520.11) was spin-coated (5000 rpm, 60 s) and baked at 80°C on a hotplate for 50 s; the final thickness was ~300 nm. The electron beam exposure dose was 240–1200 μC/cm^2^ depending on the shape of the collapsible nanopillars (ELIONIX, ELS-7800, 80 kV, 50 pA). After exposure, the AR-N resist was developed in a specific developer solution (Allresist, AR-300 47) for 80–100 s at room temperature and then rinsed with DI water for 1 min. After the development process, 10-nm gold (Au) was deposited by electron-beam evaporation (KVT, KVE-ENS 4004) to realize plasmonic nanogap structures.

### Raman measurement

Raman measurements were performed with a Renishaw inVia microRaman system. Samples were excited by a 632.8 nm He–Ne laser through a ×100 (NA = 1.25) objective. The Raman band of a silicon wafer at 520 cm^−1^ was used to calibrate the spectrometer. For 2D Raman mapping, an automatic XY stage allowed the samples to be moved in small steps of 0.2 μm.
